# Adaptive evolution of antioxidase-related genes in hypoxia-tolerant mammals

**DOI:** 10.3389/fgene.2024.1315677

**Published:** 2024-04-25

**Authors:** Qiu-Ping Wang, Chao-Yang Luo, Xiong-Hui Xu, Wen-Xian Hu, Yu-Lin Gai, You-Jing Gong, Yuan Mu

**Affiliations:** ^1^ Institute of Eastern-Himalaya Biodiversity Research, Dali University, Dali, Yunnan, China; ^2^ Erhai Watershed Ecological Environment Quality Testing Engineering Research Center of Yunnan Provincial Universities, Erhai Research Institute, West Yunnan University of Applied Sciences, Dali, Yunnan, China; ^3^ Colledge of Life Science, China West Normal University, Nanchong, Sichuan, China; ^4^ The Provincial Innovation Team of Biodiversity Conservation and Utility of the Three Parallel Rivers Region from Dali University, Dali, Yunnan, China

**Keywords:** hypoxia tolerance, antioxidase-related genes, adaptive evolution, convergent/parallel, mammals

## Abstract

To cope with the damage from oxidative stress caused by hypoxia, mammals have evolved a series of physiological and biochemical traits, including antioxidant ability. Although numerous research studies about the mechanisms of hypoxia evolution have been reported, the molecular mechanisms of antioxidase-related genes in mammals living in different environments are yet to be completely understood. In this study, we constructed a dataset comprising 7 antioxidase-related genes (*CAT*, *SOD1*, *SOD2*, *SOD3*, *GPX1*, *GPX2*, and *GPX3*) from 43 mammalian species to implement evolutionary analysis. The results showed that six genes (*CAT*, *SOD1*, *SOD2*, *SOD3*, *GPX1*, and *GPX3*) have undergone divergent evolution based on the free-ratio (M1) model. Furthermore, multi-ratio model analyses uncovered the divergent evolution between hypoxic and non-hypoxic lineages, as well as various hypoxic lineages. In addition, the branch-site model identified 9 positively selected branches in 6 genes (*CAT*, *SOD1*, *SOD2*, *SOD3*, *GPX2*, and *GPX3*) that contained 35 positively selected sites, among which 31 positively selected sites were identified in hypoxia-tolerant branches, accounting for 89% of the total number of positively selected sites. Interestingly, 65 parallel/convergent sites were identified in the 7 genes. In summary, antioxidase-related genes are subjected to different selective pressures among hypoxia-tolerant species living in different habitats. This study provides a valuable insight into the molecular evolution of antioxidase-related genes in hypoxia evolution in mammals.

## 1 Introduction

Adaptation to extreme environments is an important research field in the molecular ecology, physiological ecology, and evolutionary biology of animals. Organisms usually face environmental challenges, such as low oxygen, aridity, high ultraviolet radiation, low pressure, and extreme temperatures, of which hypoxia-tolerant species adapt to poor oxygen (O_2_) levels in aquatic, terrestrial highland, or cave environments (Nathaniel et al., 2015). Hypoxia tolerance is the adaptive ability of organisms to cope with acute and chronic hypoxia with the production of reactive oxygen species (ROS) (Ramirez et al., 2007). ROS play key roles in the body, such as signaling between cells, immunity, and participation in normal physiological reactions (Nose, 2000; Dröge, 2002), but excessive ROS produce certain oxidative damage pressure on the body (Kaelin, 2005). Consequently, numerous species have evolved various antioxidant mechanisms, including non-enzymatic and endogenous antioxidants, to adapt to peroxidative damage in hypoxic environments (Tian et al., 2021a). Among these mechanisms, the antioxidant system is crucial for counteracting the effects of hypoxic environments in hypoxia-tolerant animals. It is primarily composed of superoxide dismutases (SODs), catalase (CAT), glutathione peroxidases (GPXs), glutathione S-transferases (GSTs), and other constituents (Dröge, 2002; Wilhelm Filho et al., 2002; Birben et al., 2012).

Mammals are a highly diverse group distributed across different habitats worldwide. Many lineages inhabit hypoxic environments, including aquatic and terrestrial. Aquatic hypoxia-tolerant species can be categorized as semi-aquatic (e.g., pinnipeds) or fully aquatic (e.g., cetaceans), and terrestrial hypoxia-tolerant species primarily inhabit caves (e.g., mole rats) and high-altitude regions (e.g., yak). Previous studies showed that the activity of antioxidases is significantly higher in fully aquatic cetaceans than in terrestrial mammals (Dröge, 2002; Birben et al., 2012). In addition, semi-aquatic elephant seals (*Mirounga leonina*) also exhibited higher antioxidant enzyme activities than terrestrial mammals (Wilhelm Filho et al., 2002). In some terrestrial hypoxia-tolerant species, the antioxidase activity has also shown a similar trend, such as the activity of CAT and SOD being significantly higher in the heart and liver of Tibetan pigs than in DLY pigs (the Duroc × (Landrace × Yorkshire) hybrid pig) (Hu et al., 2022), as well as upward levels of Cu/Zn-SOD, Mn-SOD, and CAT in naked mole rats (*Heterocephalus glaber*) (Pamenter, 2022).

Researchers have uncovered some related genes or gene families that play a role in the adaptive evolutionary mechanisms of hypoxia-tolerant mammals. Tian et al. (2021b) found that the *SOD* gene family has undergone adaptive evolution in cetaceans. Additionally, gene duplication of *GPX*s occurred, and peroxidase gene families (*PRDX1* and *PRDX3*) expanded in the cetacean lineage (Yim et al., 2014; Zhou et al., 2018; Tian et al., 2021a). In addition, the genomes of the Tibetan antelope (*Pantholops hodgsonii*) showed that 247 positively selected genes are related to hypoxia tolerance, and some of them are connected with antioxidant pathways (Ge et al., 2013). Moreover, marine mammals and highland animals have evolved a convergent capacity to modulate their metabolic levels to adapt to hypoxic environments by regulating the expression of genes associated with hypoxia adaptation (Bigham et al., 2013). Generally, several studies have extensively examined the antioxidant evolutionary mechanisms among hypoxia-tolerant species. However, we need further research to understand the evolutionary pattern of different hypoxia-tolerant mammalian lineages, such as divergent evolution.

To provide further molecular evidence for the adaptive evolution of antioxidase systems in hypoxia-tolerant mammals, seven antioxidase-related genes (*CAT*, *SOD1*, *SOD2*, *SOD3*, *GPX1*, *GPX2*, and *GPX3*) of mammals in different habitats were used to perform an *in silico* analysis to solve the following scientific issues: 1) whether the antioxidase-related genes have different evolutionary patterns and 2) whether convergent evolution of antioxidase-related genes occurs in hypoxia-tolerant mammals living in diverse environments. Our study provides insights into the genetic mechanisms underlying hypoxia tolerance adaptations in mammals.

## 2 Materials and methods

### 2.1 Sequence acquisition

A total of 43 mammalian species (including hypoxia-tolerant and non-hypoxia-tolerant mammals) were selected, which covered major groups, such as Cetartiodactyla, Carnivora, Primates, and Rodentia. The gene sequences of most species were obtained from the NCBI (https://www.ncbi.nlm.nih.gov/), and the sequences of some species were obtained through the NCBI genome BLAST (genome information given in Supplementary Table S1); the sequence of the bowhead whale (*Balaena mysticetus*) was obtained from the website http://www.bowhead-whale.org/. The coding sequences were aligned by using webPRANK (https://www.ebi.ac.uk/goldman-srv/webprank/) (Löytynoja and Goldman, 2010). We corrected the multiple sequence alignment (MSA) in MEGA 7 by eyes (Kumar et al., 2016).

### 2.2 Selection pressure analysis

Selection pressure analysis is mainly based on the ratio ω of nonsynonymous (*d*_N_) to synonymous substitution (*d*_S_) of coding sequences, which is used as the main basis for detecting natural selection: ω > 1, = 1, or <1 indicates that the gene is subjected to positive, neutral, or purifying selection, respectively. The ω value was evaluated using the CodeML program in the PAML 4.9 package (Yang, 2007). The likelihood ratio test statistic (2ΔL), which approximates a chi-square (χ^2^) distribution, was used to compare nested likelihood models. Positively selected signals were identified using BEB analysis with posterior probabilities (PPs) of ≥0.8 (Yang et al., 2005). We used the TimeTree database (http://www.timetree.org/) (Kumar et al., 2017) and a previous study (Murphy et al., 2004) to obtain the mammalian phylogenetic tree for subsequent analysis (Supplementary Figure S1).

#### 2.2.1 Detection of positively selected sites

We performed site modeling (M8 and M8a) using the CodeML program in the PAML package to detect sites under positive selection (Swanson et al., 2003; Yang, 2007). To detect whether the positively selected sites were restricted to specific branching lineages, the strict branch-site model was used to detect the positive selection of specific sites that may affect specific branches in the datasets (Zhang et al., 2005).

#### 2.2.2 Branch model analysis

To understand whether the adaptive evolution of antioxidase-related genes occurs in different branches, the free-ratio (M1) model was used to examine the evolutionary rate of each branch and compared with the one-ratio model (M0), which allows only a single ω ratio for all branches.

To analyze these differences, branch models were employed using the “two-ratio (2ω),” “three-ratio (3ω),” and “five-ratio (5ω)” models, which were implemented in CodeML (Yang, 1998) (Supplementary Figure S1A–C). First, to test whether different selective pressures act on the hypoxia-tolerant and non-hypoxia-tolerant mammalian lineages, the one-ratio model that enforces the same ω ratio for all lineages was compared with the 2ω model that allows one ω ratio for all hypoxia-tolerant mammal branches and another for all remaining branches (non-hypoxia-tolerant species) (Supplementary Figure S1A). Second, to explore the rate variation between terrestrial and aquatic hypoxia-tolerant species, we used the 3ω model, which assumes independent ω values for terrestrial and aquatic hypoxia-tolerant species and the remaining lineages (Supplementary Figure S1B). Finally, the 5ω model was used to detect the differences in evolutionary rates among high-altitude, cave, fully aquatic, and semi-aquatic hypoxia-tolerant species, which was compared with the 3ω model (Supplementary Figure S1C).

### 2.3 Parallel/convergent evolution analysis

**FIGURE 1:**
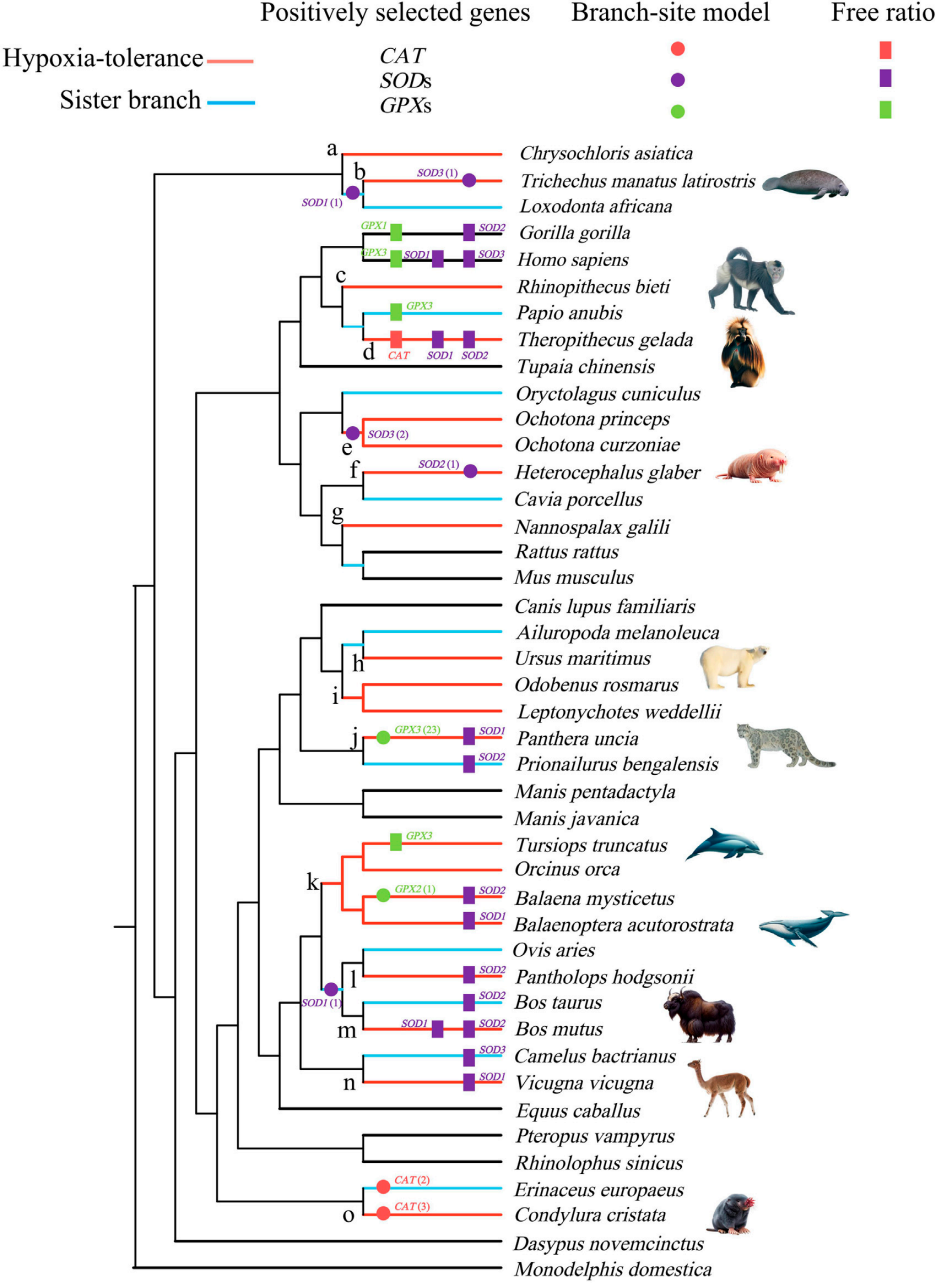
Positively selected lineages of catalase (*CAT*), superoxide dismutase (*SOD*), and glutathione peroxidase (*GPX*) genes identified by the free-ratio (rectangle) and branch-site model (circle). Branches a–o in the tree were used for the detection of convergent/parallel amino acid substitutions. The numbers in brackets behind the circle label in the tree represent the number of positively selected sites. All images are obtained from the Animal Diversity website (https://animaldiversity.org/).

To investigate whether convergent adaptation was undergone in antioxidase genes among related hypoxic branches, a parallel/convergent amino acid substitution analysis was performed. First, the ancestral amino acid sequences in each gene dataset were reconstructed using the M0 model approach implemented in the CodeML program of the PAML package (Yang, 2007). We then identified parallel/convergent amino acid replacement sites between branches with convergent hypoxia tolerance (branches a–o, Figure 1). Subsequently, CONVERGE 2 was used to test whether the parallel/convergent substitutions observed in the focal branches were fixed randomly or through natural selection (Zhang and Kumar, 1997). A statistical test was conducted to compare the observed number of parallel/convergent amino acid substitutions against the expected value. When *p* < 0.05, it meansthe convergent/parallel substitution have been driven by natural selection, rather than random mutation.

### 2.4 Protein three-dimensional structure analysis

To gain an insight into the functional implications of the detected positively selected sites, as well as the parallel/convergent amino acid sites, we mapped these sites to the three-dimensional (3D) structures of each related protein downloaded from the AlphaFold website (https://alphafold.com/) using PyMOL (DeLano, 2002), while the GPX protein structures were predicted using the I-TASSER website (https://zhanggroup.org/I-TASSER/) (Zhang, 2008). Human CAT, SODs, and GPXs were regarded as query for the analysis. Functional information about the sites and domains of each candidate protein was obtained from the UniProt website (http://www.uniprot.org/) (UniProt Consortium, 2019) and InterPro (https://www.ebi.ac.uk/interpro/) (Paysan-Lafosse et al., 2023).

## 3 Results

### 3.1 Selection pressure for antioxidase genes of hypoxia-tolerant mammals

The site model results showed that M8 was significantly better than the neutral model M8a only in the *SOD1* gene, and 7 positively selected sites were detected (12, 40, 42, 46, 47, 53, and 64) (PP > 0.80) (Supplementary Table S2).

The M0 model showed that the ω values of *CAT*, *SOD1*, *SOD2*, *SOD3*, *GPX1*, *GPX2*, and *GPX3* genes were 0.124, 0.261, 0.115, 0.129, 0.073, 0.062, and 0.132, respectively (Supplementary Table S3). In the free-ratio model, six genes (*CAT, SOD1*, *SOD2*, *SOD3*, *GPX1*, and *GPX3*) were significantly better than that in the M0 model. A total of 20 branches were detected whose ω values were significantly greater than 1, 11 of which were branches of hypoxia-tolerant species. Most positive signals were distributed in the *SOD1* and *SOD2* genes (Figure 1, Supplementary Table S4).

To evaluate whether positive selection acts on specific sites in hypoxia-tolerant species (branches) and their sister branches, the much stricter branch-site model was used. The results showed that 9 positively selected branches contained 35 positively selected sites in 6 genes (*CAT*, *SOD1*, *SOD2*, *SOD3*, *GPX2*, and *GPX3*). Of the 9 branches, 6 were hypoxia-tolerant, containing 31 positively selected sites that accounted for 89% of the total (Figure 1, Supplementary Table S5). *CAT* exhibits positive selection along the star-nosed mole (*Condylura cristata*) and hedgehog (*Erinaceus europaeus*), with three and two positively selected sites, respectively. *SOD1* exhibited positive selection in the Florida manatee (*Trichechus manatus latirostris*) and African elephant (*Loxodonta africana*) ancestors and the lineages leading to the ancestors of sheep and cattle, with one positively selected site. Moreover, evidence for positive selection was also identified in *SOD2*, along with the naked mole rat (*H. glaber*), which contained only one positively selected site. *SOD3* best fits the alternative model, along with the Florida manatee branch and the last common ancestor branch of *Ochotona curzoniae* and *Ochotona princeps*, accounting for one and two positively selected sites, respectively. *GPX2* exhibited positive selection in the bowhead whale, with one positively selected site. However, *GPX3* had 23 positively selected sites in the snow leopard (*Panthera uncia*) (PP > 0.8) (Figure 1, Supplementary Table S5).

### 3.2 Multiple ratio analyses of different hypoxia lineages

The 2ω model was used to explore whether there is evolutionary discrepancy between hypoxia-tolerant and non-hypoxia-tolerant lineages, and the results showed that the models exhibited significance in the *CAT*, *SOD1*, and *GPX2* genes. The 3ω model was used to verify the evolutionary differences in hypoxia tolerance of related genes between terrestrial and aquatic species, and the results showed significance in *SOD2* and *GPX3* genes. In addition, the ω ratio for aquatic hypoxia-tolerant mammals was much higher than that of terrestrial environment hypoxia-tolerant mammals. Based on the 5ω model, only *SOD1* exhibited model significance among various hypoxic environments (Table 1).

**TABLE 1:** Log-likelihood and omega values estimated under different branch models according to seven genes.

Genes	Model	-lnL	np	LRT	Comparisons	*p*-value	ω value		
							Non-hypoxia-tolerant mammals	Terrestrial hypoxia-tolerant mammals	Aquatic hypoxia-tolerant mammals
*CAT*	1ω	15,735.439	86				0.124	0.124	0.124
	2ω	15,733.058	87	4.763	2ω vs. 1ω	0.029	0.118	0.147	0.147
	3ω	15,732.816	88	0.484	3ω vs. 2ω	0.487	0.118	0.141	0.161
	5ω	15,730.316	90	4.999	5ω vs. 3ω	0.082	0.118	0.103/0.162	0.179/0.125
*SOD1*	1ω	5,726.588	86				0.261	0.261	0.261
	2ω	5,724.447	87	4.282	2ω vs. 1ω	0.039	0.281	0.198	0.198
	3ω	5,724.313	88	0.267	3ω vs. 2ω	0.605	0.282	0.186	0.218
	5ω	5,718.805	90	11.017	5ω vs. 3ω	0.004	0.280	0.079/0.274	0.291/0.129
*SOD2*	1ω	5,981.584	84				0.115	0.115	0.115
	2ω	5,981.059	85	1.050	2ω vs. 1ω	0.305	0.110	0.134	0.134
	3ω	5,978.089	86	5.939	3ω vs. 2ω	0.015	0.111	0.102	0.243
	5ω	5,977.952	88	0.276	5ω vs. 3ω	0.871	0.111	0.112/0.097	0.261/0.202
*SOD3*	1ω	9,905.292	86				0.129	0.129	0.129
	2ω	9,904.900	87	0.784	2ω vs. 1ω	0.376	0.134	0.119	0.119
	3ω	9,904.296	88	1.209	3ω vs. 2ω	0.272	0.134	0.110	0.141
	5ω	9,902.997	90	2.598	5ω vs. 3ω	0.273	0.133	0.093/0.124	0.160/0.094
*GPX1*	1ω	6,408.047	86				0.073	0.073	0.073
	2ω	6,407.484	87	1.126	2ω vs. 1ω	0.289	0.070	0.084	0.084
	3ω	6,407.248	88	0.473	3ω vs. 2ω	0.491	0.070	0.089	0.070
	5ω	6,405.224	90	4.047	5ω vs. 3ω	0.132	0.070	0.088/0.088	0.044/0.146
*GPX2*	1ω	4,011.799	86				0.062	0.062	0.062
	2ω	4,009.106	87	5.387	2ω vs. 1ω	0.020	0.053	0.087	0.087
	3ω	4,007.588	88	3.037	3ω vs. 2ω	0.081	0.053	0.072	0.135
	5ω	4,006.917	90	1.342	5ω vs. 3ω	0.511	0.053	0.059/0.079	0.162/0.086
*GPX3*	1ω	6,738.610	84				0.132	0.132	0.132
	2ω	6,738.067	85	1.086	2ω vs. 1ω	0.297	0.127	0.149	0.149
	3ω	6,733.463	86	9.207	3ω vs. 2ω	0.002	0.128	0.110	0.250
	5ω	6,730.568	88	5.790	5ω vs. 3ω	0.055	0.128	0.138/0.088	0.333/0.129

5ω model: in the terrestrial hypoxia-tolerant mammal group, the left number is the ω value for high-altitude hypoxia-tolerant mammals, and the right number is the ω value for cave hypoxia-tolerant mammas; in the aquatic hypoxia-tolerant mammal group, the left number is the ω value for fully aquatic hypoxia-tolerant mammals, and the right number is the ω value for semi-aquatic hypoxia-tolerant mammals.

### 3.3 Convergent evolutionary mechanisms of hypoxia-tolerant lineages

A total of 64 parallel amino acid substitution sites and 1 convergent amino acid substitution site were detected in the hypoxia-tolerant branches of the 7 genes (Table 2). The CONVERGE 2 analysis showed *p* < 0.05. In addition, most of the parallel amino acid substitution sites were distributed in the branches, leading to tolerance to terrestrial hypoxia. In the high-altitude hypoxia-tolerant branches (branches d, e, j, l, and m), we detected 6 parallel amino acid substitution sites in three genes (*SOD3*, *GPX1*, and *GPX3*); 17 parallel amino acid substitution sites were detected in cave branches (a, g, f, and o) among 7 genes (*CAT*, *SOD1*, *SOD2*, *SOD3*, *GPX1*, *GPX2*, and *GPX3*). At high altitudes (branches c, d, e, and j) and in cave species (branches a, f, g, and o), 17 parallel amino acid substitution sites were detected in 6 genes (*CAT*, *SOD1*, *SOD2*, *SOD3*, *GPX1*, and *GPX3*). Only one convergent amino acid substitution site was detected in the *SOD1* gene between the high-altitude and cave branches (branch a vs. e). Moreover, between fully aquatic mammals (branch b) and high-altitude species (branch e), we identified four parallel amino acid substitution sites in three genes (*CAT*, *SOD3*, and *GPX1*). In the high-altitude and semi-aquatic species, four parallel amino acid substitution sites were detected in four genes (*SOD1*, *SOD3*, *GPX1*, and *GPX2*). Among the cave species (branches a, f, g, and o) and fully aquatic mammals (branches b and k), nine parallel amino acid sites were detected in five genes (*CAT, SOD1*, *SOD3*, *GPX1*, and *GPX3*). In the semi-aquatic (branch i) and cave species (branches a, g, and o), four parallel amino acid substitution sites were detected in three genes (*CAT*, *SOD3*, and *GPX1*). Three parallel amino acid substitution sites in the semi-aquatic (branch i) and fully aquatic species (branches b and k) were identified in three genes (*SOD3*, *GPX1*, and *GPX3*) (Figure 1; Table 2).

**TABLE 2:** 65 parallel/convergent amino acid substitutions sites in 7 genes in hypoxia-tolerant lineages.

Branches	Genes	Sites	AA change	Observed number	Expected number	*p*-value
a vs. b	*GPX3*	216	R-Q	1	0	0
a vs. e	*SOD1*	26	T/N-S	3	0	0
	*GPX1*	201	G-E			
	*GPX3*	42	E-D			
a vs. f	*SOD3*	60	A-G	4	0	0
	*GPX1*	28	A-T			
	*GPX2*	14	I-V			
	*GPX3*	213	Y-H			
a vs. g		41	I-V	7	0	0
		42	M-L			
	*CAT*	423	T-A			
		449	K-Q			
		513	V-M			
	*SOD2*	57	A-T			
	*SOD3*	107	E-Q			
a vs. i	*SOD3*	215	C-S	1	0	0
a vs. j	*GPX3*	42	E-D	1	0	0
a vs. o	*CAT*	293	P-Q	4	0	0
		379	Y-F			
	*SOD1*	152	A-T			
	*GPX2*	126	Y-H			
b vs. e	*CAT*	381	A-T	4	0	0
	*SOD3*	76	A-E			
		161	Y-H			
	*GPX1*	54	C-G			
b vs. f	*CAT*	42	M-L	2	0	0
		455	Q-E			
b vs. g	*SOD3*	161	Y-H	2	0	0
		163	A-P			
b vs. i	*SOD3*	161	Y-H	2	0	0
	*GPX1*	204	C-S			
b vs. o	*CAT*	254	S-A	2	0	0
	*GPX1*	204	C-S			
c vs. f	*GPX1*	16	P-A	1	0	0
c vs. g	*GPX1*	137	A-V	1	0	0
c vs. o	*CAT*	159	I-L	2	0	0
	*GPX1*	89	A-T			
d vs. e	*SOD3*	57	G-D	1	0	0
d vs. f	*GPX1*	16	P-A	1	0	0
e vs. f	*CAT*	423	T-S	3	0	0
		427	G-A			
		502	A-T			
e vs. g	*CAT*	256	E-U	5	0	0
		502	A-T			
	*SOD2*	11	R-G			
	*SOD3*	161	Y-H			
	*GPX1*	199	S-A			
e vs. i	*SOD3*	161	Y-H	2	0	0
	*GPX2*	43	T-S			
e vs. j	*GPX3*	42	E-D	2	0	0
		203	V-I			
e vs. l	*SOD3*	57	G-D	1	0	0
e vs. m	*SOD3*	57	G-D	1	0	0
e vs. o	*CAT*	93	R-K	1	0	0
f vs. g	*GPX2*	92	G-S	1	0	0
f vs. k	*CAT*	508	A-T	2	0	0
	*SOD1*	96	N-E			
f vs. o	*CAT*	226	N-K	1	0	0
g vs. i	*SOD3*	161	Y-H	1	0	0
h vs. j	*SOD1*	36	T-R	1	0	0
i vs. j	*GPX3*	204	S-N	1	0	0
i vs. k	*GPX3*	192	I-V	1	0	0
i vs. o	*CAT*	436	N-D	2	0	0
	*GPX1*	204	C-S			
l vs. m	*GPX1*	204	C-A	1	0	0

### 3.4 3D structure analysis of antioxidase-related proteins

**FIGURE 2:**
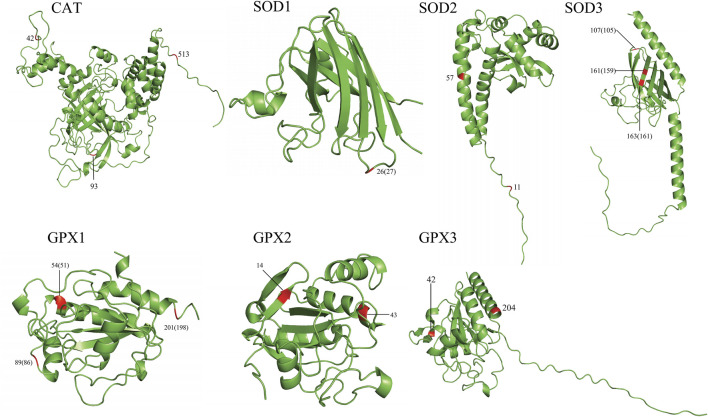
Annotation of the parallel/convergent amino acid sites of the seven genes on the 3D protein structure. The first number is for the order of analysis sequences used in this study. The numbers in brackets are for the true order of query sequences.

To gain an insight into the functional significance of the positively selected sites identified by the branch-site model and the parallel/convergent amino acid sites, we mapped these sites onto 3D structures (Figure 2, Supplementary Figure S2). In total, 21/35 positively selected sites and 19/65 convergent/parallel amino acid sites were located on important functional domains. For example, in CAT, we found that the positively selected site 444 was located next to the NADPH-binding site (445, 446). The convergent/parallel amino acid site 42 was located at the beta-strand position, site 93 was located at the turn position, and site 513 was located close to the modified residue site (511, 515). In SOD1, we found that the positively selected site 23 was located on many functionally important domains, such as the beta-strand and disulfide bond, close to the phosphoserine modification sites. Site 76 was located at the disulfide bond position. The convergent/parallel amino acid site 26 was simultaneously located at the beta-strand positions and close to the phosphoserine modification site. In SOD2, we found that the positively selected site 52 was simultaneously located on the helix, Mn/Fe-SOD-N-terminal, and next to the binding site (50) and divalent metal cation (50) positions. The convergent/parallel amino acid site 11 was located on the transport peptide and next to the natural variation site (10), whereas site 57 was located next to the modified residue site (58). In SOD3, we found that the positively selected site 87 was located on the SOD-Cu/Zn-binding domain, and site 185 was a Cu^2+^-binding site and was located next to the active site (181). These two sites and site 191 were located at the disulfide bond positions. The convergent/parallel amino acid site 107 was located close to the glycosylation modification site and located at the disulfide bond position, and sites 161 and 163 were located at the disulfide bond location. Sites 107, 161, and 163 were located on the beta-strand position. In GPX1, we found that the convergent/parallel amino acid site 54 was located at the helical position and located next to the active site (49) and the catalytic residue site (49); site 88 was located next to the modified residue site (89) and the dimer interface site (89); and site 201 was located at the modified residue site. In GPX2, we found that the convergent/parallel amino acid site 43 was located on the glutathione peroxidase active site, and site 14 was located at the beta-strand position. Additionally, most of the positively selected sites (12/23) in the GPX3 are located on crucial domains; most are located on the beta-strand and helix positions, such as sites 72, 132, 154, and 167. In addition, we found that the positively selected sites were 71, 72, 74, and 105, and these sites were close to the active sites, dimer interfaces, and catalytic residue sites. The convergent/parallel amino acid sites and residues 42 and 204 of GPX3 were all in the helix position (Figure 2; Supplementary Figure S2; Supplementary Tables S6 and S7).

## 4 Discussion

### 4.1 Evolutionary patterns of antioxidase-related genes in hypoxia-tolerant lineages

Hypoxia-tolerant mammals have evolved adaptive mechanisms, including oxygen binding, storage and transportation capacities (Ramirez et al., 2007; Michael Panneton, 2013), energy metabolism (Tian et al., 2017), and other related tissue modifications, physiological and biochemical adaptations (Avivi et al., 2010; Helbo and Fago, 2012), and associated molecular mechanisms (Tian et al., 2019; Tian et al., 2021b) to effectively cope with hypoxic environments. As an important defense barrier, the antioxidant enzyme system plays a key role in resisting oxidative damage caused by low oxygen levels. Higher SOD activity has been observed in diving species (Miller, 2012), and a significantly higher evolutionary rate of *SOD* was also identified in cetaceans (Tian et al., 2021a). In addition, positive selection affects several *GST* genes in divergent taxonomic groups, which is indicative of pervasive adaptive evolution (Tian et al., 2019). In this study, our analyses showed that antioxidase-related genes have been subjected to adaptive evolution in hypoxia-tolerant mammals. The M0 model showed that the ω values of seven genes were significantly less than 1 (ω_min_ = 0.062 for *GPX2*, ω_max_ = 0.261 for *SOD1*), indicating that purifying selection played an important role in maintaining their function of antioxidase-related genes. Based on the M1 model, six genes exhibited significant differences (Supplementary Table S4), suggesting the divergent evolutionary rates of antioxidase-related genes across all lineages in response to diverse environments. Furthermore, the branch-site model test revealed the number of positively selected sites in antioxidase genes among the hypoxia-tolerant mammals or branches. Except for the *GPX1* gene, branches subjected to positive selection were detected in six other genes, in which the number of positively selected sites accounted for 89% of the hypoxia-tolerant branches/species (Figure 1, Supplementary Table S5). Our results suggested that the core mechanisms of the adaptive evolution of antioxidase-related genes might have different patterns among different hypoxia-tolerant mammals. Antioxidase-related genes have evolved during adaptation to different hypoxic environments to facilitate their effective defense against ROS.

Mammals inhabiting diverse hypoxic environments may evolve into distinct selection pressures, even in the same lineage. For instance, cetaceans that have adapted to different diving depths have demonstrated different rates of evolution for *HBA*, *HBB*, and other associated genes (Tian et al., 2016). Hypoxia-tolerant animals exhibit remarkable adaptations in antioxidant mechanisms. For example, many marine mammals (e.g., cetaceans, pinnipeds, and manatees) can tolerate long-term hypoxia, and their recycling process of regaining oxygen after diving leads to increased ROS levels (Ramirez et al., 2007; Hermes-Lima et al., 2015). A similar tendency was detected among the antioxidase-related genes in this study. The evolutionary rates of *CAT*, *SOD1*, and *GPX2* genes in hypoxia-tolerant species were significantly higher than those in non-hypoxia-tolerant species under the 2ω model, which indicated that antioxidant-related genes have gone through accelerated evolution in adapting to the hypoxic environments. Moreover, we hypothesized that physiological or genetic differences exist in the antioxidant mechanisms between terrestrial hypoxia-tolerant and aquatic hypoxia-tolerant species due to environmental heterogeneity. The 3ω model showed that *SOD2* and *GPX3* evolved at significantly lower rates in terrestrial hypoxic environments (plateau and caves) than in aquatic environments, which suggested that the environmental differences between terrestrial and aquatic would lead to different evolutionary patterns. Meanwhile, the higher evolutionary rates in aquatic lineages might be due to the complex aquatic environments. These results implied that the evolution of related genes would evolve to adapt to different microenvironments. Thus, we hypothesized that evolutionary patterns may have evolved in antioxidation-related genes among these typical hypoxic habits. Our speculation was confirmed in the *SOD1* gene based on the 5ω model, but the specific mechanism needs to be further investigated. SOD1 is essential for the regulation and protection of cellular oxidative stress by promoting NADPH via the GAPDH/SOD1 signaling axis, its main element for the elimination of ROS (Iuchi et al., 2007; Inoue et al., 2010; Montllor-Albalate et al., 2022). In this study, the evolutionary rates of *SOD1* genes vary significantly in different environments, which might be related to the different adaptive abilities of mammals to respond to oxidative stress in different hypoxic environments. In summary, the evolutionary rate of antioxidase-related genes in hypoxia-tolerant mammals varies in different habitats.

### 4.2 Parallel/convergent evolution of hypoxia-tolerant lineages

Hypoxia tolerance is a classic example of convergent evolution. Although hypoxia-tolerant species inhabit different environments and have different modes of adaptation to hypoxia, inherent similarities in hypoxia tolerance may drive recurrent evolution at the sequence level, which has manifested as convergent or parallel amino acid changes (Li et al., 2010). For example, echolocation in toothed whales, insect-eating bats, and pigtail mice (*Typhlomys cinereus*) (Shen et al., 2012; Parker et al., 2013; He et al., 2021) and convergent or parallel amino acid site substitutions have been detected in different hypoxic environments (Tian et al., 2017). In this study, genes with convergent evolution were also detected on the branches leading to hypoxia tolerance. For example, two parallel amino acid substitution sites were identified between the ancestral branches of the plateau pika and blind mole rat (*Nannospalax galili*) in *CAT* (branch e vs. g, Figure 1), and one parallel amino acid substitution site was identified between the naked mole rat (branch f) and cetacean (branch k) (Figure 1). Notably, 21 parallel amino acid substitution sites were detected in the *CAT* gene, accounting for 32% of the total parallel/convergent sites. Efficient scavenging of hydrogen peroxide (H_2_O_2_) is an important measure for mammals to cope with hypoxic environments during hypoxic adaptation (Baker et al., 2023), and CAT is the most important enzyme for organisms to catabolize hydrogen peroxide to enable the scavenging of oxygen radicals (Scibior and Czeczot, 2006; Kirkman and Gaetani, 2007; Ighodaro and Akinloye, 2018). Accordingly, hypoxia-tolerant species may strongly converge through hydrogen peroxide scavenging. Moreover, parallel/convergent substitutions in *GPX* genes were detected in almost all hypoxia-tolerant branches. *GPX*s are crucial in inhibiting lipid peroxidation processes (Ighodaro and Akinloye, 2018), suggesting a general convergence of hypoxia-tolerant species in inhibiting oxidative damage to lipids.

In particular, some functional sites also have evolved into convergence. For example, the convergent/parallel amino acid site 26 of SOD1 is located close to the phosphoserine residue modification site. Studies have shown that phosphoserine residues can affect secondary protein structures and alter their biological activities and catalytic functions (Shi, 2009). Site 107 of SOD3 is located close to the glycosylation modification site that may play an important role in the modification of translational and post-translational proteins (Staudacher et al., 2022), suggesting that site 107 may alter the antioxidant activity of SOD3. Studies have shown that all SODs require a catalytic metal (Cu or Mn) for their activation (Fukai and Ushio-Fukai, 2011; Skopp et al., 2019). In summary, hypoxia-tolerant species have evolved convergent antioxidant mechanisms to adapt to their respective hypoxic environments due to convergent adaptation.

### 4.3 Functional analysis of positively selected sites

Positively selected amino acid sites are labeled on the 3D structure of proteins. Most of these sites are located at or next to the important functional sites and domains, which might affect the structure and function of proteins, reflecting their key role in the adaptation to hypoxia. For example, the positively selected site detected by the branch-site model is in CAT, site 444 is located next to the NADPH-binding sites (445 and 446), and studies have shown that NADPH can effectively protect catalase against H_2_O_2_ (Kirkman et al., 1999). The result suggested that site 444 may play an important role in the antioxidant activity of CAT. In addition, site 52 of SOD2 is located next to the Mn/Fe-SOD-N-terminal and close to the metal cation (50), site 87 of SOD3 is located on the SOD-Cu/Zn-binding domain, and site 185 is located on the Cu^2+^-binding site, suggesting that these sites play an important role in the catalytic reaction of antioxidase. We also observed that some sites were located on or next to the active sites or dimer interface residues (associated with ligand binding and dimerization) (Epp et al., 1983; Maiorino et al., 1998), such as site 23 of SOD1, sites 87 and 185 of SOD3, site 89 of GPX1, site 43 of GPX2, and sites 72 and 74 of GPX3. This may alter the binding ability or catalytic activity of SOD and GPX to the substrate.

In summary, these findings must be validated by functional studies in future research studies. Therefore, it is necessary to conduct more studies on hypoxia-tolerant species and antioxidase-related genes to better understand the adaptive mechanisms of hypoxia-tolerant mammals.

## 5 Conclusion

Hypoxia tolerance is a complex and interesting evolutionary adaptation, and its study at the molecular level is conducive to revealing the effects of extreme hypoxic environments. Our results indicate that antioxidase-related genes have undergone different evolutionary trajectories in hypoxia- and non-hypoxia-tolerant mammals, as well as in various hypoxic environments among hypoxia-tolerant mammals. Multiple convergent/parallel amino acid substitution sites have been detected among hypoxia-tolerant mammalian lineages, and these sites were located on or next to the functional site, providing molecular evidence for the convergent mechanism of hypoxia adaptation in mammals. Our results further revealed the molecular mechanism of antioxidase-related genes involved in the adaption to hypoxia in hypoxia-tolerant mammals.
