# Supplementary material for: Adaptive evolution of antioxidase-related genes in hypoxia-tolerant mammals
Source: Front Genet. 2024 Apr 25;15:1315677. doi: 10.3389/fgene.2024.1315677 (PMC11079137; doi:10.3389/fgene.2024.1315677)
Supplement: Supplementary file 1 [file Table1.docx]

**Supplementary Table 1** List of genes accession numbers used in this study

| **Order** | **Family** | **Common name** | **Scientific name** | ***CAT*** | ***SOD1*** | ***SOD2*** | ***SOD3*** | ***GPX1*** | ***GPX2*** | ***GPX3*** |
| --- | --- | --- | --- | --- | --- | --- | --- | --- | --- | --- |
| Primates | Cercopithecidae | Black snub-nosed monkey | *Rhinopithecus bieti* | XM_017849719.1 | XM_017883889.1 | XM_017853513.1 | XM_017866756.1 | XM_017868084.1 | XM_017892430.1 | XM_017875756.1 |
|  |  | Gelada baboon | *Theropithecus gelada* | XM_025356343.1 | XM_025380341.1 | XM_025383712.1 | XM_025386576.1 | XM_025375856.1 | XM_025392263.1 | XM_025387542.1 |
|  |  | Olive baboon | *Papio anubis* | XM_003910050.4 | XM_003895543.3 | XM_009206338.3 | XM_021938334.2 | XM_003894429.4 | XM_031668048.1 | XM_009209421.2 |
|  | Hominidae | Human | *Homo sapiens* | NM_001752.4 | NM_000454.5 | NM_000636.4 | NM_003102.4 | NM_000581.4 | NM_002083.4 | NM_002084.5 |
|  | Pongidae | Western gorilla | *Gorilla gorilla* | XM_004050923.3 | XM_004062687.2 | XM_031011830.1 | XM_004038497.3 | XM_031008954.1 | XM_004055307.3 | XM_004042839.3 |
| Rodentia | Muridae | Mouse | *Mus musculus* | NM_009804.2 | NM_011434.2 | NM_013671.3 | NM_011435.3 | NM_008160.6 | NM_030677.2 | NM_008161.4 |
|  |  | Rat | *Rattus rattus* | XM_032903812.1 | XM_032900460.1 | XM_032894729.1 | XM_032916859.1 | XM_032910620.1 | XM_032907978.1 | XM_032912589.1 |
|  | Caviidae | Guinea pig | *Cavia porcellus* | NM_001172925.1 | XM_003467248.4 | XM_003466367.4 | XM_003467399.4 | XM_003476448.4 | XM_005004774.3 | XM_003464498.4 |
|  | Bathyergidae | Naked mole rat | *Heterocephalus glaber* | XM_004852060.3 | XM_013071287.2 | XM_004869025.3 | XM_004847205.3 | XM_004834027.3 | XM_004837393.3 | XM_004836654.3 |
|  | Spalacidae | Blind mole rat | *Nannospalax galili* | XM_008852360.3 | XM_008835989.3 | XM_008833859.3 | XM_008837328.3 | XM_008853188.3 | XM_008852130.2 | XM_008851876.3 |
| Lagomorpha | Ochotonidae | Pateau pika | *Ochotona curzoniae* | XM_040980081.1 | XM_040980729.1 | XM_040965266.1 | XM_040966174.1 | XM_040994463.1 | XM_040975449.1 | XM_040967087.1 |
|  |  | Pika | *Ochotona princeps* | XM_004585411.2 | XM_004588624.2 | XM_004595461.3 | XM_004579339.1 | XM_004581757.2 | XM_004584476.1 | XM_004587526.2 |
|  | Leporidae | Rabbit | *Oryctolagus cuniculus* | XM_002709045.3 | NM_001082627.2 | XM_051854201.1 | XM_051828779.1 | NM_001085444.1 | NM_001256893.1 | NM_001256900.1 |
| Scandentia | Tupaiidae | Treeshrew | *Tupaia chinensis* | XM_006162101.3 | XM_006155261.3 | XM_006152239.3 | KB320878.1 | XM_006172131.2 | XM_006142408.3 | XM_006150834.2 |
| Cetartiodactyla | Bovidae | Wild yak | *Bos mutus* | XM_005890889.2 | XM_005893360.2 | XM_014479669.1 | XM_014478628.1 | XM_005909375.2 | XM_005909763.2 | XM_005897329.2 |
|  |  | Domestic cattle | *Bos taurus* | NM_001035386.2 | NM_174615.2 | NM_201527.2 | NM_001082610.1 | NM_174076.3 | NM_001163139.2 | NM_174077.5 |
|  |  | Tibetan antelope | *Pantholops hodgsonii* | NW_005816935.1 | NW_005813146.1 | NW_005816019.1 | NW_005815902.1 | NW_005812141.1 | NW_005810114.1 | NW_005804064.1 |
|  |  | Sheep | *Ovis aries* | XM_004016396.5 | NM_001145185.2 | NM_001280703.1 | XM_042251270.1 | XM_004018462.5 | XM_004010720.5 | XM_015096153.3 |
|  | Camelidae | Vicugna | *Vicugna vicugna* | MU059884.1 | MU060713.1 | MU060312.1 | MU060072.1 | MU061775.1 | MU059059.1 | MU062225.1 |
|  |  | Bactrian camel | *Camelus bactrianus* | XM_010964693.2 | XM_010956595.2 | XM_010965766.2 | XM_010963047.2 | XM_010969258.2 | XM_010962266.2 | XM_010969999.2 |
|  | Balaenopteridae | Minke whale | *Balaenoptera acutorostrata* | XM_007181052.1 | XM_007183692.2 | XM_007186105.2 | XM_007168445.1 | XM_007198435.2 | XM_007184291.2 | XM_007188628.2 |
|  | Balaenidae | Bowhead whale | *Balaena mysticetus* | bmy_18415T0 | bmy_11579T0 | bmy_19635T0 | bmy_02070T0 | bmy_06005T0 | bmy_17476T0 | bmy_10532T0 |
|  | Delphinidae | Killer whale | *Orcinus orca* | XM_004263991.1 | XM_004264510.4 | XM_004278885.4 | XM_004266200.4 | XM_004286798.3 | XM_004262132.3 | XM_004280338.4 |
|  |  | Bottlenose dolphins | *Tursiops truncatus* | XM_033860462.1 | XM_004322979.3 | XM_019951789.2 | NC_047038.1 | XM_004332180.2 | XM_033851053.1 | XM_033853485.1 |
| Perissodactyla | Equidae | Horse | *Equus caballus* | XM_001914718.5 | NM_001081826.3 | NM_001082517.2 | XM_005615084.3 | NM_001166479.1 | NM_001166481.1 | NM_001115158.1 |
| Carnivora | Felidae | Leopard cat | *Prionailurus bengalensis* | XM_043580918.1 | XM_043593751.1 | XM_043592682.1 | XM_043572866.1 | XM_043590384.1 | XM_043556365.1 | XM_043600998.1 |
|  |  | Snow leopard | *Panthera uncia* | XM_049648439.1 | XM_049627936.1 | XM_049654532.1 | XM_049630391.1 | XM_049640460.1 | XM_049611488.1 | XM_049611488.1 |
|  | Ursidae | Polar bear | *Ursus maritimus* | XM_040638941.1 | XM_040621040.1 | XM_040628017.1 | XM_040625215.1 | XM_040628814.1 | XM_008700888.2 | XM_040630394.1 |
|  |  | Giant panda | *Ailuropoda melanoleuca* | XM_002920612.4 | XM_002928799.4 | XM_034669402.1 | XM_034672003.1 | XM_011226672.3 | XM_011217735.3 | XM_002920757.4 |
|  | Canidae | Domestic dog | *Canis lupus familiaris* | NM_001002984.1 | NM_001003035.1 | XM_038654563.1 | XM_038662067.1 | NM_001115119.1 | NM_001115135.1 | NM_001164454.1 |
|  | Phocidae | Weddell seal | *Leptonychotes weddellii* | XM_031027893.1 | XM_031041374.1 | XM_006733221.2 | XM_006749688.2 | XM_006752386.2 | XM_006738619.2 | XM_006752411.2 |
|  | Odobenidae | Pacific walrus | *Odobenus rosmarus* | XM_004406087.2 | XM_004406294.1 | XM_004401126.2 | XM_004402858.2 | XM_004417735.2 | XM_004409373.2 | XM_004402830.2 |
| Pholidota | Manidae | Malayan pangolin | *Manis javanica* | XM_037019800.1 | XM_017662668.2 | XM_017666020.2 | XM_036994698.1 | XM_036994352.1 | XM_017660414.2 | XM_017651581.2 |
|  |  | Chinese pangolin | *Manis pentadactyla* | XM_036891658.1 | XM_036880296.1 | XM_036886688.1 | XM_036921001.1 | XM_036877789.1 | XM_036906048.1 | XM_036893404.1 |
| Chiroptera | Rhinolophidae | Rhinolophus rex | *Rhinolophus sinicus* | XM_019738543.1 | XM_019730176.1 | XM_019749569.1 | XM_019743917.1 | XM_019723860.1 | XM_019733417.1 | XM_019734325.1 |
|  | Pteropodidae | Large flying fox | *Pteropus vampyrus* | XM_023528790.1 | XM_011373845.2 | XM_011380358.2 | XM_011365246.2 | XM_011375420.2 | XM_011384386.2 | XM_011371220.2 |
| Eulipotyphla | Talpidae | Star-nosed mole | *Condylura cristata* | XM_004682933.2 | XM_004675345.2 | XM_012735174.1 | XM_004681505.2 | XM_004676172.2 | XM_004681782.2 | — |
|  | Erinaceidae | Hedgehog | *Erinaceus europaeus* | XM_007521812.2 | XM_007519700.2 | XM_007536201.2 | XM_007535118.2 | XM_007533589.2 | XM_007531725.2 | XM_007528771.2 |
| Cingulata | Dasypodidae | Nine-banded armadillo | *Dasypus novemcinctus* | XM_023586671.1 | XM_004468609.2 | XM_004449782.3 | XM_012526473.2 | XM_004484924.3 | XM_004477282.3 | XM_004472013.2 |
| Proboscidea | Elephantidae | African elephant | *Loxodonta africana* | XM_003412126.3 | XM_010590320.2 | XM_010598844.2 | XM_003411292.3 | XM_010589805.2 | XM_010588689.2 | XM_003404609.3 |
| Afrosoricida | Chrysochloridae | Golden mole | *Chrysochloris asiatica* | XM_006864883.1 | XM_006862595.1 | XM_006868463.1 | XM_006866297.1 | XM_006878221.1 | XM_006878294.1 | XM_006863891.1 |
| Sirenia | Trichechus manatus | Florida manatee | *Trichechus manatus latirostris* | NW_004443942.1 | XM_004386984.2 | — | JH594640.1 | NW_004443937.1 | NW_004443946.1 | NW_004444519.1 |
| Didelphimorphia | Didelphidae | Gray short-tailed opossum | *Monodelphis domestica* | XM_007497259.2 | XM_001365107.3 | XM_007484848.2 | XM_016423300.1 | XM_007507924.2 | XM_007473041.2 | XM_007473895.2 |
