# Supplementary figures and images for: Adaptive evolution of antioxidase-related genes in hypoxia-tolerant mammals
Source: Front Genet. 2024 Apr 25;15:1315677. doi: 10.3389/fgene.2024.1315677 (PMC11079137; doi:10.3389/fgene.2024.1315677)

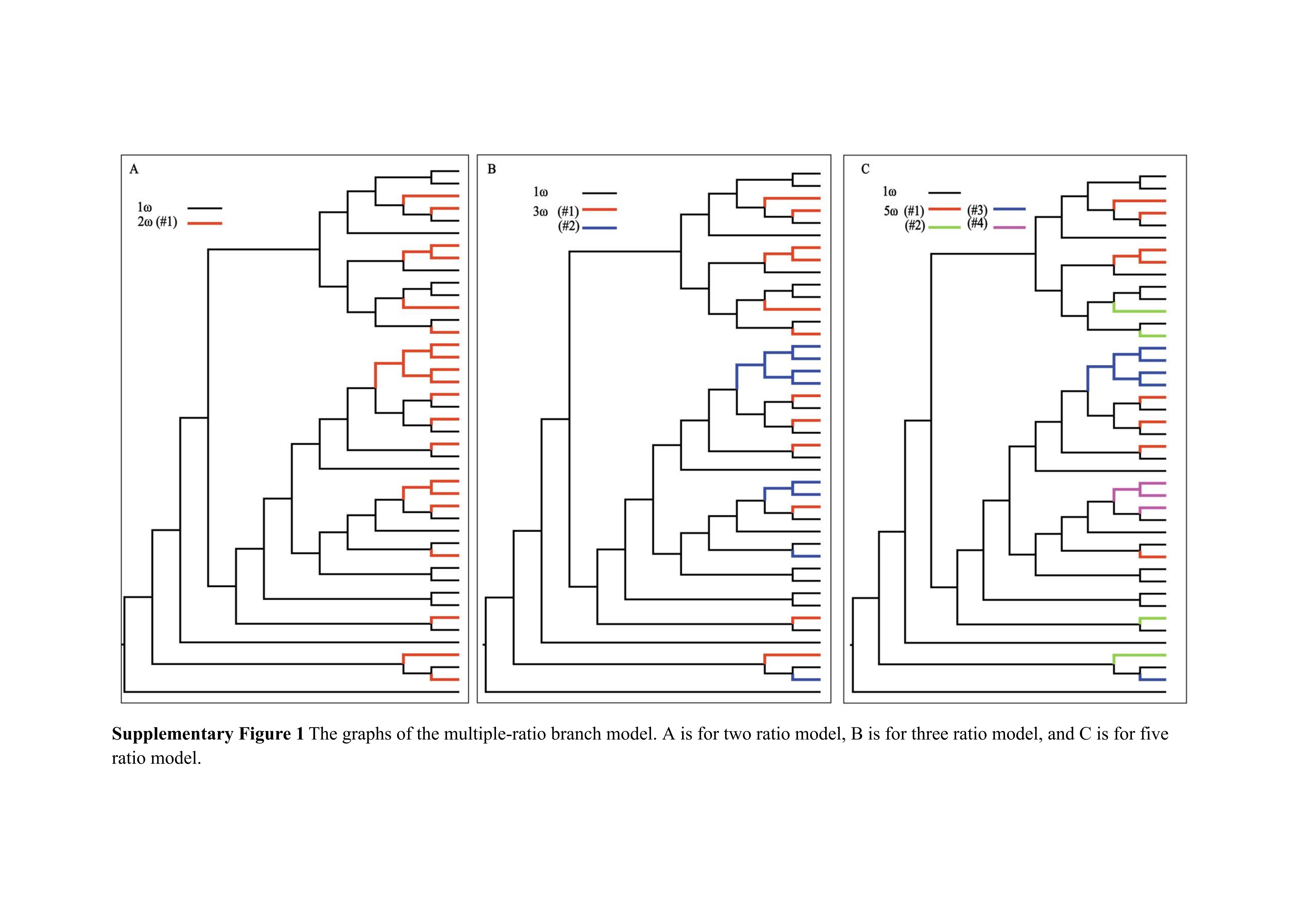

Supplement: Supplementary file 2 [file Image1.jpeg]

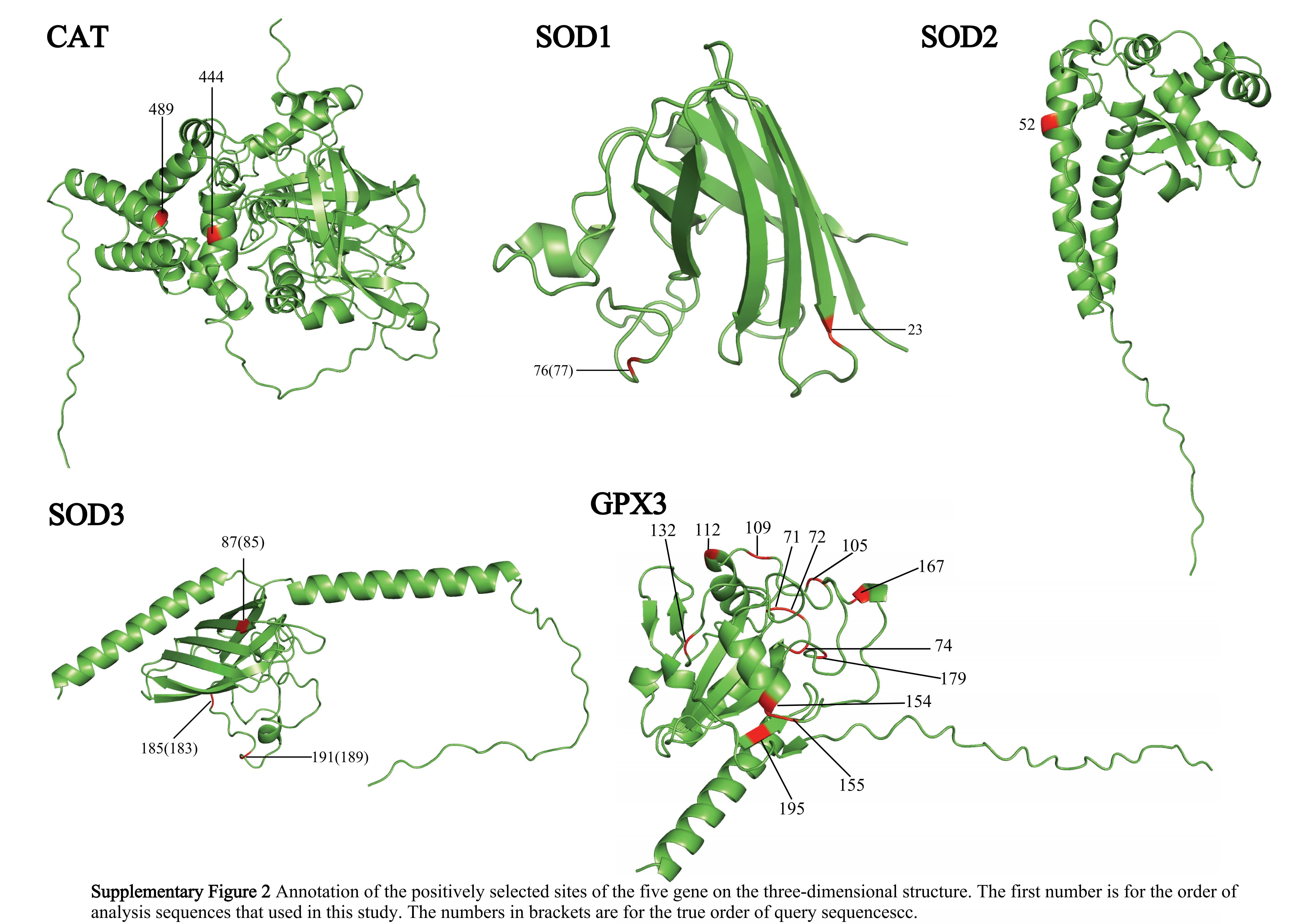

Supplement: Supplementary file 4 [file Image2.jpeg]
