# Supplementary material for: Adaptive evolution of antioxidase-related genes in hypoxia-tolerant mammals
Source: Front Genet. 2024 Apr 25;15:1315677. doi: 10.3389/fgene.2024.1315677 (PMC11079137; doi:10.3389/fgene.2024.1315677)
Supplement: Supplementary file 3 [file Table7.docx]

**Supplementary Table 7** Convergent/parallel functional site analysis (identity with sites of human)

| **Proteins** | **Accession** | **sites** | **Site feature** **(UniPprot or InterPro)** |
| --- | --- | --- | --- |
| CAT | AF-P04040-F1 | 42  93  513 | Beta-strand  Turn  Close to modified residue site (511, 515) |
| SOD1 | AF-P04179-F1 | 26 | Beta-strand; close to phosphoserine modification site |
| SOD2 | AF-P04179-F1 | 11  57 | Close to natural variant site (10); Transit peptide  Helix; close to modified residue site (58) |
| SOD3 | AF-P08294-F1 | 107  161  163 | Close to glycosylation site; beta-strand; disulfide bond  Beta-strand; disulfide bond  Beta-strand; disulfide bond |
| GPX1 | Predicted using the I-TASSER website | 54  89  201 | Close to active site (49); Helix; close to catalytic residues (49) (InterPro);  Glutathione peroxidase active site (InterPro)  Close to modified residue (88); close to dimer interface (88) (InterPro);  Close to modified residue site |
| GPX2 | Predicted using the I-TASSER website | 14  43 | Beta-strand  Helix; close to active site (40); close to glutathione peroxidase active site (InterPro) |
| GPX3 | Predicted using the I-TASSER website | 42  204 | Helix  Helix |

Numbers in parentheses are indicated as functional sites adjacent to that detected sites.
