# Supplementary material for: Adaptive evolution of antioxidase-related genes in hypoxia-tolerant mammals
Source: Front Genet. 2024 Apr 25;15:1315677. doi: 10.3389/fgene.2024.1315677 (PMC11079137; doi:10.3389/fgene.2024.1315677)
Supplement: Supplementary file 5 [file Table2.docx]

**Supplementary Table 2** Results of the site model on the *SOD1* gene

| **Gene** | **Model** | **-lnL** | **Model**  **comparison** | **2ΔlnL** | **df** | ***P*-value** | **Parameters** | **Positive site**  **(PP≥80%)** |
| --- | --- | --- | --- | --- | --- | --- | --- | --- |
| *SOD1* | M8 | 3019.894 | M8 vs M8a | 19.013 | 1 | <0.001 | 2.055  2.170  2.142  2.131  2.164  2.059  2.165 | 12 H 0.919  40 T 0.996**  42 S 0.977*  46 S 0.970*  47 H 0.991**  53 P 0.924  64 A 0.992** |
|  | M8a | 3029.400 |  |  |  |  |  |  |
