# Supplementary material for: Adaptive evolution of antioxidase-related genes in hypoxia-tolerant mammals
Source: Front Genet. 2024 Apr 25;15:1315677. doi: 10.3389/fgene.2024.1315677 (PMC11079137; doi:10.3389/fgene.2024.1315677)
Supplement: Supplementary file 6 [file Table5.docx]

**Supplementary Table 5** Selective pressure analysis of hypoxia-tolerant mammals and sister branches by branch-site model

| **Genes** | **The branch of** | **Model** | **-lnL** | **Model**  **comparison** | **2ΔlnL** | **df** | ***P*-value** | **Parameters** | **Positive site**  **(PP≥80%)** |
| --- | --- | --- | --- | --- | --- | --- | --- | --- | --- |
| *CAT* | *Condylura cristata* | Ma | 15080.923 | Ma vs Ma0 | 8.106 | 1 | 0.004 | ω0=0.041 ω1=1.000 ω2=17.840 | 426 S 0.964*  444 R 0.874  489 Y 1.000** |
|  |  | Ma0 | 15084.976 |  |  |  |  | ω0=0.041 ω1=1.000 ω2=1.000 |  |
|  | *Erinaceus europaeus* | Ma | 15088.810 | Ma vs Ma0 | 6.036 | 1 | 0.014 | ω0=0.043 ω1=1.000 ω2=998.999 | 47 R 0.965*  507 - 0.945 |
|  |  | Ma0 | 15091.828 |  |  |  |  | ω0=0.042 ω1=1.000 ω2=1.000 |  |
| *SOD1* | (*Loxodonta africana*, *Trichechus manatus latirostris*) | Ma | 5341.697 | Ma vs Ma0 | 10.372 | 1 | 0.001 | ω0 =0.032 ω1=1.000 ω2=77.085 | 76 D 0.998** |
|  |  | Ma0 | 5346.884 |  |  |  |  | ω0=0.033 ω1=1.000 ω2=1.000 |  |
|  | ((*Bos mutus*, *Bos taurus*), (*Pantholops hodgsonii*, *Ovis aries*)) | Ma | 5347.270 | Ma vs Ma0 | 4.233 | 1 | 0.040 | ω0=0.036 ω1=1.000 ω2=69.416 | 23 Q 0.974* |
|  |  | Ma0 | 5349.703 |  |  |  |  | ω0=0.034 ω1=1.000 ω2=1.000 |  |
| *SOD2* | *Heterocephalus glaber* | Ma | 5802.356 | Ma vs Ma0 | 5.526 | 1 | 0.019 | ω0=0.048 ω1 =1.000 ω2=39.540 | 52 S 0.992** |
|  |  | Ma0 | 5805.119 |  |  |  |  | ω0=0.048 ω1=1.000 ω2=1.000 |  |
| *SOD3* | *Trichechus manatus latirostris* | Ma | 9709.042 | Ma vs Ma0 | 4.125 | 1 | 0.042 | ω0=0.084 ω1=1.000 ω2=13.133 | 185 Q 0.966* |
|  |  | Ma0 | 9711.104 |  |  |  |  | ω0=0.083 ω1=1.000 ω2=1.000 |  |
|  | (*Ochotona curzoniae*, *Ochotona princeps*) | Ma | 9709.320 | Ma vs Ma0 | 4.319 | 1 | 0.038 | ω0=0.083 ω1=1.000 ω2=998.992 | 87 K 0.855  191 K 0.934 |
|  |  | Ma0 | 9711.479 |  |  |  |  | ω0=0.083 ω1=1.000 ω2=1.000 |  |
| *GPX2* | *Balaena mysticetus* | Ma | 3965.089 | Ma vs Ma0 | 9.134 | 1 | 0.003 | ω0=0.039 ω1=1.000 ω2=999.000 | 189 I 0.960* |
|  |  | Ma0 | 3969.657 |  |  |  |  | ω0=0.039 ω1=1.000 ω2=1.000 |  |
| *GPX3* | *Panthera uncia* | Ma | 6537.044 | Ma vs Ma0 | 9.728 | 1 | 0.002 | ω0=0.067 ω1=1.000 ω2=42.386 | 43 Y 0.923  71 S 0.976*  72 Y 0.923  74 L 0.980*  105 K 0.966*  108 P 0.994**  109 G 0.870  112 S 0.987*  128 V 0.992**  132 Q 0.997**  137 G 0.873  146 K 0.996**  154 S 0.998**  155 C 0.966*  158 T 0.989*  159 S 0.979*  163 G 0.940  167 R 0.933  174 K 0.950  176 H 0.995**  179 R 0.983*  195 M 0.982*  199 H 0.957* |
|  |  | Ma0 | 6541.908 |  |  |  |  | ω0=0.064 ω1=1.000 ω2=1.000 |  |
