# Supplementary material for: Adaptive evolution of antioxidase-related genes in hypoxia-tolerant mammals
Source: Front Genet. 2024 Apr 25;15:1315677. doi: 10.3389/fgene.2024.1315677 (PMC11079137; doi:10.3389/fgene.2024.1315677)
Supplement: Supplementary file 7 [file Table3.docx]

**Supplementary Table 3** One-ratio model analysis in seven genes

| **Genes** | **Model** | **-lnL** | **Model**  **comparison** | **2ΔlnL** | **df** | ***P*-value** | **Parameters** |
| --- | --- | --- | --- | --- | --- | --- | --- |
| *CAT* | A(fix omega=0) | 15735.439 | A vs B | 1160.912 | 1 | <0.001 | ω=0.124 |
|  | B(fix omega=1) | 16315.895 |  |  |  |  | ω=1 |
| *SOD1* | A(fix omega=0) | 5726.588 | A vs B | 100.514 | 1 | <0.001 | ω=0.261 |
|  | B(fix omega=1) | 5776.845 |  |  |  |  | ω=1 |
| *SOD2* | A(fix omega=0) | 5981.584 | A vs B | 493.349 | 1 | <0.001 | ω=0.115 |
|  | B(fix omega=1) | 6228.259 |  |  |  |  | ω=1 |
| *SOD3* | A(fix omega=0) | 9905.292 | A vs B | 611.974 | 1 | <0.001 | ω=0.129 |
|  | B(fix omega=1) | 10211.279 |  |  |  |  | ω=1 |
| *GPX1* | A(fix omega=0) | 6408.047 | A vs B | 935.174 | 1 | <0.001 | ω=0.073 |
|  | B(fix omega=1) | 6875.634 |  |  |  |  | ω=1 |
| *GPX2* | A(fix omega=0) | 4011.799 | A vs B | 591.499 | 1 | <0.001 | ω=0.062 |
|  | B(fix omega=1) | 4307.549 |  |  |  |  | ω=1 |
| *GPX3* | A(fix omega=0) | 6738.609 | A vs B | 458.369 | 1 | <0.001 | ω=0.132 |
|  | B(fix omega=1) | 6967.794 |  |  |  |  | ω=1 |
