# Supplementary material for: Adaptive evolution of antioxidase-related genes in hypoxia-tolerant mammals
Source: Front Genet. 2024 Apr 25;15:1315677. doi: 10.3389/fgene.2024.1315677 (PMC11079137; doi:10.3389/fgene.2024.1315677)
Supplement: Supplementary file 8 [file Table4.docx]

**Supplementary Table 4** Free-ratio model analysis of seven genes

| **Genes** | **Model** | **-lnL** | **Model**  **comparison** | **2ΔlnL** | **df** | ***P*-value** |
| --- | --- | --- | --- | --- | --- | --- |
| *CAT* | M0(one ratio) | 15735.439 | M1vsM0 | 230.781 | 83 | <0.001 |
|  | M1(free ratio) | 15620.049 |  |  |  |  |
|  |  |  |  |  |  |  |
| *SOD1* | M0(one ratio) | 5726.588 | M1vsM0 | 175.367 | 83 | <0.001 |
|  | M1(free ratio) | 5638.904 |  |  |  |  |
|  |  |  |  |  |  |  |
| *SOD2* | M0(one ratio) | 5981.584 | M1vsM0 | 128.747 | 81 | <0.001 |
|  | M1(free ratio) | 5917.211 |  |  |  |  |
|  |  |  |  |  |  |  |
| *SOD3* | M0(one ratio) | 9905.292 | M1vsM0 | 161.126 | 83 | <0.001 |
|  | M1(free ratio) | 9824.729 |  |  |  |  |
|  |  |  |  |  |  |  |
| *GPX1* | M0(one ratio) | 6408.047 | M1vsM0 | 114.020 | 83 | 0.014 |
|  | M1(free ratio) | 6351.037 |  |  |  |  |
|  |  |  |  |  |  |  |
| *GPX2* | M0(one ratio) | 4011.799 | M1vsM0 | 100.727 | 83 | 0.090 |
|  | M1(free ratio) | 3961.436 |  |  |  |  |
|  |  |  |  |  |  |  |
| *GPX3* | M0(one ratio) | 6738.609 | M1vsM0 | 149.732 | 81 | <0.001 |
|  | M1(free ratio) | 6663.744 |  |  |  |  |
