# Supplementary material for: Adaptive evolution of antioxidase-related genes in hypoxia-tolerant mammals
Source: Front Genet. 2024 Apr 25;15:1315677. doi: 10.3389/fgene.2024.1315677 (PMC11079137; doi:10.3389/fgene.2024.1315677)
Supplement: Supplementary file 9 [file Table6.docx]

**Supplementary Table 6** Branch-site model functional site analysis (identity with sites of human)

| **Proteins** | **Accession** | **sites** | **Site feature (UniProt or** **InterPro)** |
| --- | --- | --- | --- |
| CAT | AF-P04040-F1 | 444  489 | Helix; close to NADPH-binding site (445, 446) (InterPro)  Helix |
| SOD1 | AF-P04179-F1 | 23  76 | Beta-strand; close to the phosphoserine (26) and modified residue site (26);  close to E-class dimer interface (20) (InterPro)  Beta-strand; disulfide bond; close to binding site (72, 81) |
| SOD2 | AF-P04179-F1 | 52 | Helix; close to binding site (50); close to divalent metal cation (50); Mn/Fe-SOD-N-terminal |
| SOD3 | AF-P08294-F1 | 87  185  191 | Beta-strand; disulfide bond; close to P-class dimer interface (92) and E-class dimer interface (83) (InterPro); Cu-Zn-binding domain (InterPro)  Beta-strand; disulfide bond; close to Cu^2+^-binding site and active site (181) (InterPro)  Disulfide bond |
| GPX3 | Predicted using the I-TASSER website | 71  72  74  105  109  112  132  154  167  179  195 | Beta strand; close to active site (73); catalytic residue site (73) (InterPro)  Beta strand; close to active site (73); catalytic residue site (73) (InterPro)  Turn; close to active site (73); catalytic residue site (73) (InterPro)  Dimer interface and close to catalytic residue (107) (InterPro)  Close to dimer interface (InterPro)  Close to dimer interface (111, 114)  Beta-strand  Helix  Helix  Close to catalytic residue (181)  Beta-strand |

Numbers in parentheses are indicated as functional sites adjacent to that detected sites.
